# Mitochondrial damage by α-synuclein causes cell death in human dopaminergic neurons

**DOI:** 10.1038/s41419-019-2091-2

**Published:** 2019-11-14

**Authors:** Goutham K. Ganjam, Kathrin Bolte, Lina A. Matschke, Sandra Neitemeier, Amalia M. Dolga, Matthias Höllerhage, Günter U. Höglinger, Agata Adamczyk, Niels Decher, Wolfgang H. Oertel, Carsten Culmsee

**Affiliations:** 1 Institute for Pharmacology and Clinical Pharmacy, Biochemical-Pharmacological Center, University of Marburg, Marburg, Germany; 2 Department of Neurology, University of Marburg, Marburg, Germany; 3 Center for Mind, Brain and Behaviour – CMBB, Marburg, Germany; 4 Laboratory for Cell Biology I, Department of Biology, University of Marburg, Marburg, Germany; 5 Institute of Physiology and Pathophysiology, University of Marburg, Marburg, Germany; 6 Department of Molecular Pharmacology, Groningen Research Institute of Pharmacy, University of Groningen, Groningen, The Netherlands; 7 German Center for Neurodegenerative Diseases (DZNE), Munich, Germany; 8 Department of Cellular Signaling, Mossakowski Medical Research Centre, Polish Academy of Sciences, Warsaw, Poland; 9 Department of Pediatrics, The Third Affiliated Hospital of Zhengzhou University, Zhengzhou, China

**Keywords:** Cell death in the nervous system, Cellular neuroscience, Parkinson's disease

## Abstract

Evolving concepts on Parkinson’s disease (PD) pathology suggest that α-synuclein (aSYN) promote dopaminergic neuron dysfunction and death through accumulating in the mitochondria. However, the consequence of mitochondrial aSYN localisation on mitochondrial structure and bioenergetic functions in neuronal cells are poorly understood. Therefore, we investigated deleterious effects of mitochondria-targeted aSYN in differentiated human dopaminergic neurons in comparison with wild-type (WT) aSYN overexpression and corresponding EGFP (enhanced green fluorescent protein)-expressing controls. Mitochondria-targeted aSYN enhanced mitochondrial reactive oxygen species (ROS) formation, reduced ATP levels and showed severely disrupted structure and function of the dendritic neural network, preceding neuronal death. Transmission electron microscopy illustrated distorted cristae and many fragmented mitochondria in response to WT-aSYN overexpression, and a complete loss of cristae structure and massively swollen mitochondria in neurons expressing mitochondria-targeted aSYN. Further, the analysis of mitochondrial bioenergetics in differentiated dopaminergic neurons, expressing WT or mitochondria-targeted aSYN, elicited a pronounced impairment of mitochondrial respiration. In a pharmacological compound screening, we found that the pan-caspase inhibitors QVD and zVAD-FMK, and a specific caspase-1 inhibitor significantly prevented aSYN-induced cell death. In addition, the caspase inhibitor QVD preserved mitochondrial function and neuronal network activity in the human dopaminergic neurons overexpressing aSYN. Overall, our findings indicated therapeutic effects by caspase-1 inhibition despite aSYN-mediated alterations in mitochondrial morphology and function.

## Introduction

Parkinson’s disease (PD) is a neurodegenerative movement disorder characterised by the progressive dopaminergic neurodegeneration in the substantia nigra pars compacta. Aggregation of aSYN into Lewy bodies (LBs) is a hallmark of PD, dementia with LBs, and neurodegenerative synucleinopathies like multiple systems atrophy or pure autonomic failure^1^. Duplication, triplication or A30P, A53T, A53E, G51D, or E46K point mutations in the aSYN gene have been associated with autosomal-dominant forms of PD^2,3^. Transgenic animal models of PD and cellular overexpressing aSYN revealed mitochondrial localisation of aSYN may accelerate aSYN toxicity in dopaminergic neurons^4–6^. Familial forms of PD-like mutations in PTEN-induced putative kinase-1, aSYN, leucine-rich repeat kinase-2, parkin or DJ-1 have also been associated with dysfunctional mitochondria^7,8^.

Variety of cell death mechanisms were linked to the pathogenesis of PD, such as extrinsic and intrinsic apoptosis, (regulated) necrosis and autophagy^9^. Studies in Lund human mesencephalic (LUHMES) neurons identified an iron-dependent ferroptosis cell death pathway is involved in the pathogenesis of PD^10^. In programmed cell death (PCD), impaired redox balance, deregulated cellular homeostasis and death signalling pathways converge at the mitochondria. Dysfunctional mitochondria undergo fragmentation and loss of membrane potential, and enhanced reactive oxygen species (ROS) levels promoting neurodegeneration^11^. Oxidative conditions results in phosphorylation at serine 129 of aSYN, thereby initiating the formation of toxic insoluble aSYN oligomers^12^. aSYN neurotoxicity shares molecular events observed in PCD, including mitochondrial dysfunction, failure of autophagy, impaired redox balance and calcium deregulation^13–15^.

A fraction of aSYN associated with mitochondria or supraphysiological levels of aSYN interacts with endoplasmic reticulum and mitochondrial complex I impairing mitochondrial function^4,16–18^. Interactions of aSYN with mitochondria were identified in the mitochondrial fractions from the SN of PD^4^. A specific transcript of aSYN containing a long 3′-untranslated region (UTR) that was identified in PD patient’s brain tissue showed preferential accumulation of aSYN in the mitochondria^19^. These observations strongly suggest the existence of a mitochondrial variant of aSYN that may significantly contribute to dopaminergic neurodegeneration. However, studies on aSYN variants directly targeting mitochondria to investigate the dopaminergic neurodegeneration are not reported. Therefore, this study focused on the overexpression of a mitochondria-targeted aSYN and its implications on mitochondrial function and cell viability in LUHMES cells. Differentiated LUHMES dopaminergic neurons provide a validated in vitro model system to investigate the neurodegenerative mechanisms associated with aSYN toxicity^20^. By using LUHMES system, we investigated the deleterious effects of mitochondrial aSYN localisation with a particular focus on mitochondrial ultrastructure and respiratory function. Furthermore, we screened different compound classes for inhibition of aSYN-mediated mitochondrial damage and neurotoxicity. Our findings indicated that caspase activation occurs upstream of mitochondrial pathways of aSYN toxicity.

## Materials and methods

### Chemical compounds

The chemical inhibitors used to test against aSYN toxicity in this study are listed here. QVD-OPh (*N*-(2-quinolyl)valyl-aspartyl-(2,6-difluorophenoxy)methylketone) (EMD-Millipore, Billerica, MA, USA) and zVAD-FMK (*N*-benzyloxycarbonyl-Val-Ala-Asp(O-Me) fluoromethyl ketone (Enzo Life Sciences, Farmingdale, NY, USA) are broad-spectrum caspase inhibitors. For convenience from here on QVD-OPh is referred to as QVD. DHB (ethyle-3,4-dihydroxybenzoate) (Sigma-Aldrich, St. Louis MO, USA) is a HIF-PHDs (hypoxia-inducible factor prolyl hydroxylases) inhibitor. NS309 (Sigma-Aldrich, St. Louis MO, USA) is a SK channel activator and interleukin-1β-converting enzyme (ICE-1) inhibitor II or caspase-1 inhibitor, YVAD-CMK (ICE-1 is also known as caspase-1) (EMD-Millipore, Billerica, MA, USA). All the inhibitors used in this study were dissolved in dimethyl sulfoxide (DMSO) and treated LUHMES cells at a concentration of 10 µM after 24 h of AAV (adeno-associated viral vector) viral infections and DMSO was used as vehicle control. After 48 h of the drug treatment, the cells were subjected to respective analysis.

### Recombinant AAV2-aSYN viral vector engineering and virus generation

The human complementary DNA (cDNA) for WT-aSYN from pcDNA3 aSYN was excised by *Nhe*1 and *Hin*dIII restriction enzymes (Thermo Fisher Scientific, Darmstadt, Germany) and subcloned into AAV2-hSynapsin-EGFP-WPRE vector by replacing the *EGFP* (enhanced green fluorescent protein) reporter gene to obtain AAV2-hSynapsin-aSYN-WPRE^21,22^. For mitochondrial-specific overexpression of aSYN, we ligated the mitochondrial localisation sequence (MLS) of cytochrome *c* encoding nearly 3 kDa at the 5′ of aSYN cDNA to obtain AAV2-hSynapsin-mito-aSYN-WPRE. Similarly, MLS was cloned in front of EGFP to obtain AAV2-hSynapsin-mito-EGFP-WPRE vector for mitochondrial EGFP control vector. All the AAV2 viral vectors applied in this study use the human synapsin promoter to restrict the transgene expression only to neurons. The presence of a Woodchuck hepatitis virus post-transcriptional regulatory element (WPRE) enhances the stability of the messenger RNA (mRNA) and sustained transgene expression. All molecular cloning procedures were performed in SURE2 bacterial cells to minimise unwanted recombinant events. Recombinant AAV vectors of serotype 2 were produced by transfecting AAV *cis* plasmids encoding the gene of interest and a viral helper plasmid pDG2 as previously described^23^. The obtained AAV viruses are referred to as AAV2/2, where the first number determines the genotype and the second number indicates the serotype. The titre of the viruses was measured by quantifying the isolated viral genome by using quantitative PCR. For convenience reasons, the cytosolic vectors were named AAV2-hSyn-EGFP or AAV2-hSyn-aSYN and mitochondrial AAV vectors were named AAV2-hSyn-mito-EGFP or AAV2-hSyn-mito-aSYN throughout the paper.

### LUHMES and primary rat cortical neuronal cell culture

Post-mitotic differentiated human dopaminergic neuronal cells LUHMES were used in this study^24,25^. LUHMES cells were proliferated in cell culture flasks (Nunclon DELTA surface, NUNC A/S, St. Louis, MO, USA) coated with 0.1 mg/ml poly-l-lysine (PLL) (Sigma-Aldrich, St. Louis MO, USA) at +4 °C overnight. For experiments, cell culture dishes were coated with 0.1 mg/ml PLL overnight and washed three times with sterile water, followed by coating with 5 µg/ml fibronectin (Sigma-Aldrich, St. Louis MO, USA) overnight in the incubator (37 °C, 5% CO_2_). Before plating the cells, fibronectin was removed, and the wells were washed with phosphate-buffered saline (PBS) and dried. Cells were plated at a density of 55,000/cm^2^ in Dulbecco’s modified Eagle’s medium (DMEM)/F12 (Sigma-Aldrich, St. Louis MO, USA) with 1% N2-supplement (Life Technologies, Carlsbad, CA, USA), 0.04 µg/ml basic fibroblast growth factor (R&D Systems, Minneapolis, MN, USA). After 24 h of plating, the medium was exchanged to differentiation medium DMEM/F12 with 1% N2-supplement, 1 µg/ml tetracycline, 0.49 mg/ml dibutyryl cyclic AMP (Sigma-Aldrich, St. Louis MO, USA) and 2 ng/ml glial cell-derived neurotrophic factor (R&D Systems, Minneapolis, MN, USA)^26^. Following 5 days of differentiation, the cells were replenished with fresh media and infected with AAV2 viral particles at a concentration of 10^12^ genomic copies per millilitre (gc/ml). After 72 h, the cells were washed once with 1× PBS and subjected to the respective analysis. Primary rat cortical neurons were isolated from embryonic day 18 (E18) Sprague–Dawley rats and cultured as explained^27^. Two-day cultured primary cortical neurons were replenished with fresh media and infected with AAV2 viral particles at a concentration of 10^12^ gc/ml. After 72 h, the cells were washed with 1× PBS and used for the respective analysis.

### Protein extraction and Western blotting

For protein analysis, cells were briefly washed with 1× PBS and lysed with a buffer containing 0.25 M mannitol, 0.05 M Tris, 1 M EDTA (ethylenediaminetetraacetic acid), 1 M EGTA (ethylene glycol-bis(β-aminoethyl ether)-*N*,*N*,*N*′,*N*′-tetraacetic acid), 1 mM DTT (dithiothreitol), 1% Triton X (Sigma-Aldrich, St. Louis MO, USA), and supplemented with complete mini protease and phosphatase inhibitor cocktail tablets (Roche Diagnostics, Mannheim, Germany). Cell membrane fragments and other insoluble components from the lysates were pelleted by centrifugation at 13,000 × *g* for 10 min at 4 °C. Total protein amount was determined by using the Pierce BCA protein assay kit (Thermo Fisher Scientific, Darmstadt, Germany) and 30 µg of protein samples were run on a 12.5% sodium dodecyl sulfate gel and subsequently blotted onto a PVDF (polyvinylidene fluoride) membrane at 15 V for 90 min. The membranes were incubated overnight with primary rabbit anti-aSYN antibodies 1:1000 (Santa Cruz, SC-7011-R, clone-20, now discontinued) or mouse monoclonal anti-aSYN antibodies 1:1000 (Santa Cruz, SC-12767, clone-211) or 1:10,000 mouse monoclonal anti-actin antibodies (mpbio, MP-691001, clone-4) or goat anti-EGFP antibodies 1:1000 (Rockland Immunochemicals, 600-101-215M) at 4 °C. After incubation with the corresponding secondary horse radish peroxidase (HRP)-labelled antibody (Vector Laboratories, Burlingame, CA, USA) and HRP substrate (PJK GmbH, Kleinblittersdorf, Germany), chemiluminescence was detected with Chemidoc software (Bio-Rad, Munich, Germany).

### Immunocytochemistry

Differentiated human dopaminergic neurons were fixed by using 4% paraformaldehyde for 15 min and permeabilised by Triton X-100 at 0.04% as described before^28^. Primary antibodies against human aSYN (Santa Cruz C20; Santa Cruz Biotechnology, Dallas, USA), β3-tubulin (Invitrogen, Karlsruhe, Germany) at a concentration of 1:200 were incubated overnight at 4 °C, followed by secondary anti-rabbit, anti-mouse antibodies coupled to fluorophore Dyelight 488, and Dyelight 649 (Invitrogen) at a concentration of 1:500. Images were obtained by using a CCD camera (DFC 360, FX, Leica, Wetzlar, Germany) connected to an epifluorescent microscope (DMI 6000 B, Leica). For co-localisation studies, cells were co-stained with MitoTracker Deep Red (200 ng/ml) for mitochondria and DAPI (4′,6-diamidino-2-phenylindole, 1 µg/ml) for the nucleus. Quantification and analysis of neuronal network was performed by using the Image J software.

### Cell viability

Cell viability was quantified by the reduction of MTT (3-(4,5-dimethylthiazol-2-yl)-2, 5-diphenyltetrazolium bromide) by the LUHMES cells for 1 h in the cell culture incubator. Total medium containing MTT was removed, and the cell culture dishes were frozen at −80 °C for at least an hour to stop the reaction. After thawing at room temperature, the dye was dissolved in 80 µl of DMSO, and the absorbance was measured at 570 nm with a reference filter at 630 nm with a FLUOStar OPTIMA reader (BMG Labtech, Ortenberg, Germany) as described^29^.

Additionally, to analyse the cell viability at different time intervals, we also measured the lactate dehydrogenase (LDH) release in the media after 24, 48, and 72 h of viral infections by using commercially available LDH-Glo^TM^ Cytotoxicity assay (J2380).

### ATP measurement

ATP levels in human dopaminergic neurons upon aSYN overexpression were analysed by luminescence detection according to the manufacturer’s protocol from ViaLight TM plus kit (Lonza, Verviers, Belgium). LUHMES cells were cultured and differentiated on white 96-well plates (10,000 cells/well, Greiner Bio-one, Frickenhausen, Germany). After 72 h of aSYN expression, ATP levels were analysed by luminescence detection using a FLUOstar OPTIMA reader (BMG Labtech, Offenburg, Germany).

### Mitochondrial membrane potential analysis

Changes in the mitochondrial membrane potential in human dopaminergic neurons upon aSYN-induced toxicity were analysed by staining the cells with TMRE (tetramethylrhodamine ethyl ester) dye (Invitrogen, Karlsruhe, Germany). Cells were cultured and differentiated on PLL/fibronectin-coated coverslips in 24-well plates and infected with respective AAV viruses. After 72 h, cells were incubated with culture medium containing 200 nM TMRE dye for 30 min at 37 °C. Cells were washed briefly with PBS and imaged immediately with an epifluorescence microscope at an excitation wavelength of 620 and 670 nm for emission (red). Cells harbouring red fluorescing mitochondria were considered healthy and quantified from at least 500 cells per condition from three independent experiments without the knowledge of treatment history, and the control in each individual experiment was set as 100%.

### Mitochondrial ROS analysis

Generation of mitochondrial ROS in response to aSYN toxicity was analysed by staining the LUHMES cells with MitoSOX Red (Invitrogen, Karlsruhe, Germany). LUHMES cells cultured on PLL/fibronectin-coated coverslips in 24-well plates were infected with respective AAV2 viruses for 48 h. Cells were incubated with medium containing 200 nM MitoSOX red for 30 min at 37 °C. Then, the cells were gently washed with PBS and imaged immediately using an epifluorescence microscope at a wavelength of 620 nm excitation and 670 nm emission (red). Quantification of ROS producing, that is, red fluorescing mitochondria from at least 500 cells per condition was performed from three independent experiments without the knowledge of the treatment history, and the control in each individual experiment was set as 100%.

### Electrophysiology

Electrical activity of human dopaminergic neurons was measured by on-cell and whole-cell patch-clamp recordings using an Axopatch 200B amplifier (Molecular Devices, Sunnyvale, CA, USA) and Clampex 10.0 software (Molecular Devices, Sunnyvale CA, USA). Patch pipettes were prepared from borosilicate glass capillaries GB 150TF-8P (Science Products, Hofheim, Germany), and had tip resistances between 3 and 5 MΩ. Cells were continuously superfused with an extracellular solution consisting of (in mM): 138 NaCl, 5.6 KCl, 1.2 MgCl_2_, 2.6 CaCl_2_, 25 d-glucose, and 5 HEPES (pH 7.4 adjusted with NaOH). Whole-cell patch-clamp experiments were conducted with an internal solution containing (in mM): 10 NaCl, 76 K_2_SO_4_, 10 KCl, 1 MgCl_2_, and 5 HEPES (pH 7.35 with KOH). Recordings of spontaneous action potentials were obtained in the on-cell voltage clamp configuration, and membrane potential recordings were performed in the whole-cell current clamp configuration. Data were digitised at 10 kHz with a Digidata 1440A digitiser (Molecular Devices, Sunnyvale CA, USA) and filtered at 1–5 kHz. Electrode capacitance was compensated, and only cells with series resistances lower than 15 MΩ were recorded. Data were analysed with ClampFit10 (Molecular Devices, Sunnyvale, CA, USA).

### Oxygen consumption rate determination

Changes in mitochondrial respiration function of human dopaminergic neurons upon aSYN expression were analysed using the XF^e^96 analyser from Seahorse Bioscience (Agilent Technologies, Waldbronn, Germany). LUHMES cells were differentiated on PLL/fibronectin-coated XF^e^96-well microplates (10,000 cells/well) and infected with the AAV2-aSYN variants and EGFP as respective controls for 72 h. Cells were briefly washed with growth medium, and the medium was replaced before the measurements with 180 µl of assay medium (containing 4.5 g/l glucose, 2 mM glutamine, 1 mM pyruvate, pH 7.35). Cells with assay medium were incubated at 37 °C for 60 min. Three baseline measurements were recorded before applying the different compounds. Oligomycin was added in port A (20 µl) at a final concentration of 3 µM, in port B 22.5 µl of FCCP (carbonylcyanide-4-(trifluoromethoxy)-phenylhydrazone) at a final concentration of 0.4 µM and in port C 25 µl of rotenone/antimycin A at a final concentration of 1 µM. Three measurements were performed after the addition of each compound with 4 min mixing intervals, followed by 3 min measuring periods.

### Active caspase staining

Caspase activation in LUHMES cells attributed to aSYN toxicity was analysed by FITC-labelled FITC-VAD-FMK in situ staining of active caspases. Following 72 h of AAV2 viral infection, cells were incubated with medium containing 5 µM of FITC-VAD-FMK (G7461, Promega, Madison, USA) for 30 min at 37 °C. Cells were briefly washed with PBS and imaged immediately with an epifluorescence microscope. Quantification of active caspase intensities was performed by using the Image J software from at least three individual experiments.

### Transmission electron microscopy

Mitochondrial ultrastructure of human dopaminergic neurons was analysed by transmission electron microscopy (TEM). Cells were cultured and differentiated on micro sapphire plates coated with 0.1 mg/ml and 1 µg/ml fibronectin for AAV2 viral infections. Following 48 h of viral infection, cells were cryo-immobilised on the sapphire plates by high-pressure freezing (HPF Compact 02, Engineering Office M. Wohlwend GmbH, Sennwald, Switzerland). Subsequently, the cells were freeze substituted with acetone in combination with 2% OsO_4_, 0.25% uranyl acetate, and 5% H_2_O^30–32^. Freeze substitution was carried out in the automated AFS2 unit (Leica Microsystems GmbH, Wetzlar, Germany) at −90 °C for 22 h, −60 °C for 8 h, −30 °C for 8 h, and then held at 0 °C for 2 h. The heating time between each step was 1 h. After washing the samples two times in ice-cold acetone, they were gradually infiltrated in Epon 812 resin at room temperature, followed by polymerisation at 60 °C for 3 days. Ultrathin sections of embedded samples were collected on uncoated nickel grids (400 square mesh). For immunolocalisation studies, cells were labelled with primary antibody against aSYN (Santa Cruz C20; Santa Cruz Biotechnology, Dallas, USA) and secondary antibodies were coupled to 20 nm gold particles. Labelling procedure was performed as explained earlier^32^. Transmission electron micrographs were recorded with a JEOL TEM 2100 microscope operated at 120 kV equipped with a fast-scan 2 K × 2 K CCD camera F214 (TVIPS, Gauting, Germany).

### Statistics

All data were calculated as means ± standard error of the mean (SEM). Analysis of variance (ANOVA) was performed followed by Scheffé’s post hoc test for statistical comparisons between multiple groups. Calculations were analysed using the WinStat standard statistical software (R. Fitch Software, Bad Krozingen, Germany). Statistical significant differences were assumed at *p < 0.05, **p < 0.01, or ***p < 0.001.

## Results

### Disturbed ultrastructural integrity of mitochondria by aSYN in differentiated LUHMES neurons

**Fig. 1:**
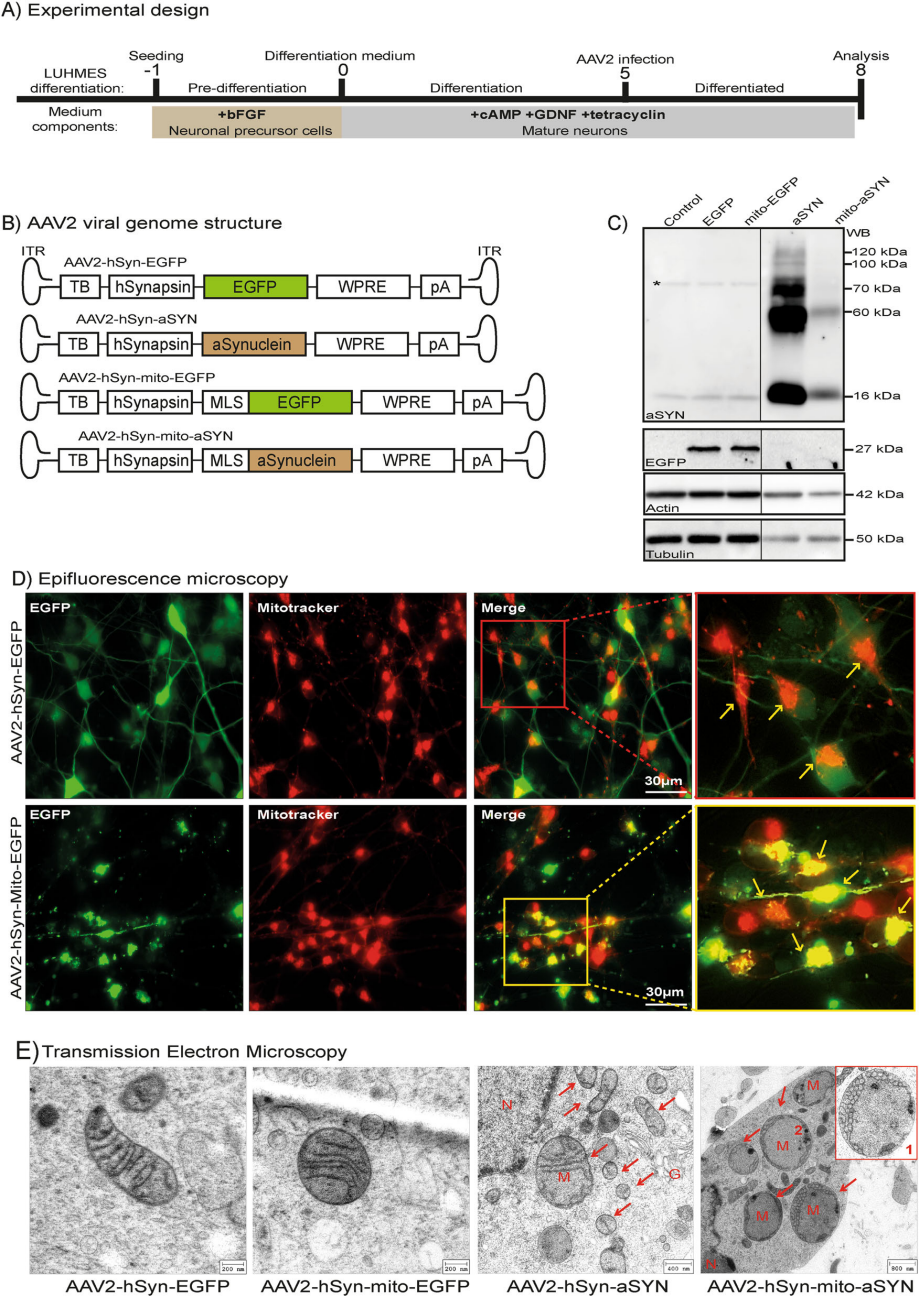
Altered mitochondrial structural integrity by aSYN expression in LUHMES neurons. **a** Schematic representation of the human dopaminergic neuron differentiation and aSYN overexpression as a model system of PD. Days are numbered relative to the seeding (day −1), differentiation (day 0), AAV viral infection (day 5) and analysis (day 8). **b** Cartoons depicting AAV2 viral vector genomes harbouring respective DNA elements. TB, transcription blocker; hSynapsin, human synapsin promoter; EGFP, enhanced green fluorescent protein; WPRE, woodchuck hepatitis virus post-transcriptional regulatory element; pA, poly A transcription stop signal; MLS, mitochondrial localisation signal from cytochrome *c*; aSynuclein, human wild-type aSYN; ITR, inverted terminal repeats. **c** Western blot analysis of AAV2 viral-mediated EGFP, mitochondrial EGFP, aSYN and mitochondrial aSYN in differentiated LUHMES cells. * indicates unspecific band present in all conditions. For convenience, Western blot image was cut and rearranged in the order of treatment as depicted and the unaltered Western blot image was presented in the Supplementary Fig. 1**a**. **d** Representative epifluorescent images of differentiated LUHMES cells expressing cytosolic EGFP (first panel) and mitochondrial EGFP (second panel) following infection with AAV2-hSyn-EGFP and AAV2-hSyn-mito-EGFP. **e** Transmission electron microscopy (TEM) depicting well-preserved mitochondrial cristae structures in differentiated human dopaminergic LUHMES neurons expressing EGFP controls. Neurons infected with WT-aSYN-expressing AAV2 depicted many fragmented mitochondria marked by red arrows. Mitochondrial outer and inner cristae membranes were deformed (M). Neurons expressing mitochondria-targeted aSYN identified many massively swollen mitochondria, and cristae structure was utterly deformed and cornered to the periphery as small circular structures shown in inset (Box 1). (G, Golgi apparatus; M, mitochondria; N, nucleus)

LUHMES neurons were differentiated to dopaminergic phenotype as explained earlier^20^ and infected with the AAV2 viruses for 3 days for the intended analysis (experimental scheme Fig. 1a). AAV2-mediated expression of wild-type aSYN (aSYN) and mitochondria-targeted aSYN (mito-aSYN) in LUHMES neurons were first analysed by Western blotting. LUHMES neurons expressing WT-aSYN showed a band at 16 kDa representing the monomer of aSYN. We observed aSYN at higher molecular weights corresponding to 55–60, 70 and at 100–120 kDa despite denaturing conditions indicating the formation of toxic aSYN oligomers (Fig. 1c). Interestingly, neurons expressing mito-aSYN depicted two bands corresponding to the monomer size and a 60 kDa band representing the only oligomer observed (Fig. 1c). Western blots showed that the formation of oligomers by aSYN occurs predominantly in the cytoplasm and the assembly patterns differ from the mito-aSYN.

We then tested the AAV2-mediated expression of WT-aSYN and mito-aSYN or EGFP controls in the dopaminergic neurons by immunocytochemistry and fluorescence imaging. To detect mitochondrial localisation of aSYN or EGFP, AAV2-transduced neurons were stained with MitoTracker deep red before fluorescence imaging. Fluorescence microscopy of neurons infected with AAV2-hSyn-mito-EGFP virus revealed that the expression of EGFP was exclusively restricted to the mitochondria (Fig. 1d, second panel). AAV2-hSyn-EGFP-transduced neurons showed ubiquitous expression of EGFP throughout the cell body including the neurites (Fig. 1d, first panel). Immunocytochemistry analysis of aSYN in neurons transduced with AAV2-hSyn-mito-aSYN identified co-localisation of aSYN with MitoTracker red-stained mitochondria (Supplementary Fig. 1b, third panel), while AAV2-hSyn-aSYN-mediated aSYN expression was detected throughout the cell body and the neurites (Supplementary Fig. 1b, second panel). Non-infected control cells show only endogenous aSYN expression and did not co-localise in the mitochondria (Supplementary Fig. 1b, first panel). These results confirmed the functionality of AAV2 viruses and demonstrated that both mitochondria-targeted aSYN and EGFP constructs restricted their expression to the mitochondria of dopaminergic neurons.

Investigations in post-mortem brain tissue identified the mitochondrial presence of aSYN in the dopaminergic neurons of PD patients^4^. The direct influence of aSYN on mitochondrial structure and integrity in human dopaminergic neurons is unknown. With our new AAV system for expression of MLS-tagged aSYN or WT-aSYN, we investigated the effects of aSYN on the ultrastructural integrity of mitochondria in human dopaminergic neurons by electron microscopy. Control LUHMES neurons or EGFP-expressing neurons showed intact mitochondria with well-preserved double membranes and cristae structures (Fig. 1e, Supplementary Fig. 1c). However, neurons overexpressing WT-aSYN showed many fragmented mitochondria and depicted few or disorganised cristae structures (Fig. 1e). However, neurons expressing mito-aSYN displayed massively swollen mitochondria with severely disturbed cristae structure (Fig. 1e, right panel). In the cell bodies of mito-aSYN-expressing neurons, the mitochondrial cristae structures were totally disorganised and appeared as small vesicle-like structures assembled at the periphery of the organelle (Fig. 1e, Box 1). These findings indicated pronounced structural damage to the mitochondria by the mito-aSYN.

To validate whether the structural abnormalities could be attributed to the mitochondrial localisation of aSYN, we performed immunoelectron microscopy using immunogold labelling of aSYN. In TEM analysis of the neurons expressing WT-aSYN, we identified the presence of immunogold particles in the cytoplasm (Supplementary Fig. 2a, Box 1), at the nuclear membrane (Supplementary Fig. 2a, Box 2) and at the mitochondrial membrane (Supplementary Fig. 2b, Box 3). Neurons expressing mitochondria-targeted aSYN revealed exclusive localisation of immunogold particles in the inner mitochondrial membrane confirming accumulation of mito-aSYN variant within the mitochondria (Supplementary Fig. 2c, Box 4). These results demonstrate that aSYN targets mitochondria and, when accumulating or overexpressing mito-aSYN severely disturbs the structural integrity of these organelles.

### Mitochondrial dysfunction induced by aSYN in LUHMES neurons

**Fig. 2:**
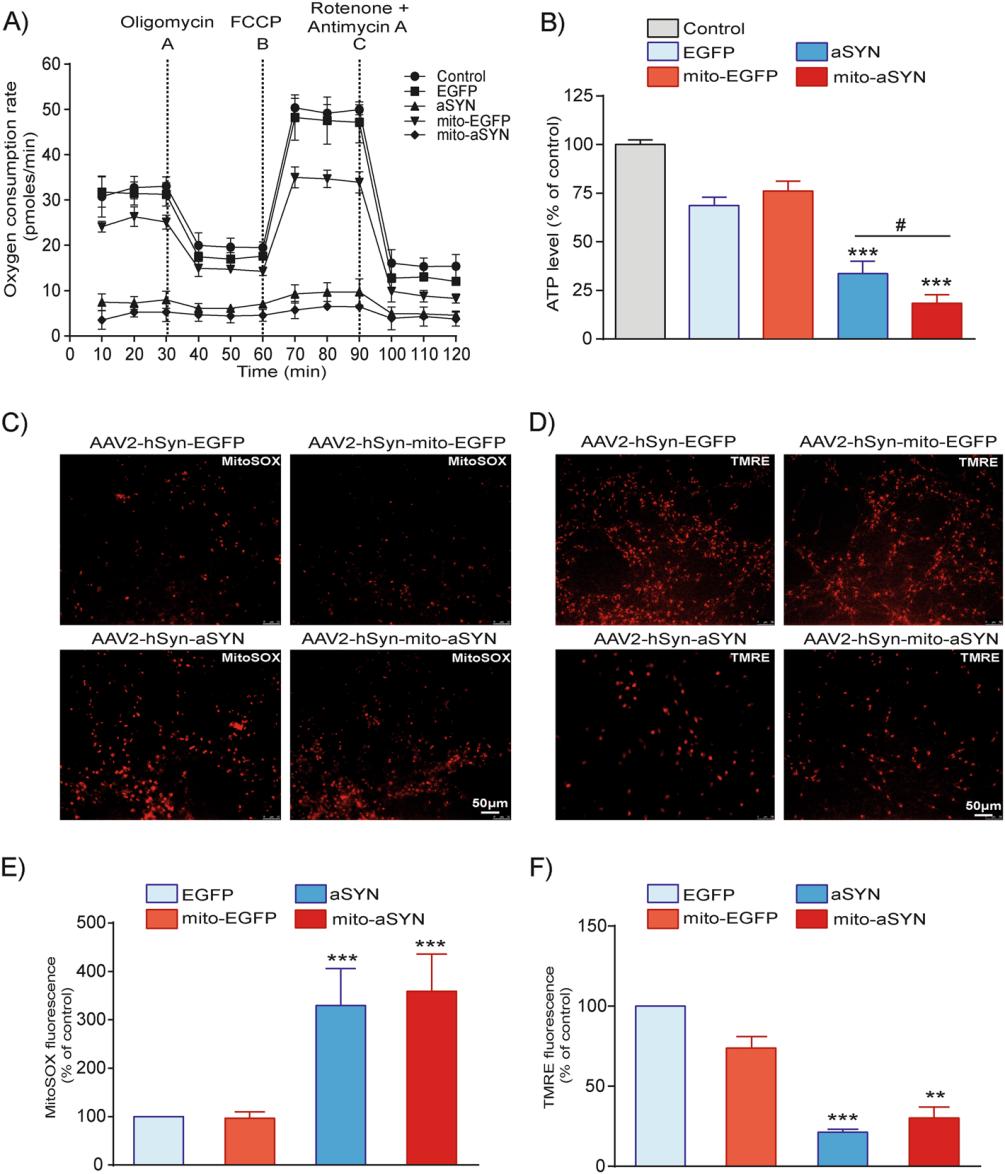
Expression of aSYN impairs mitochondrial function in LUHMES neurons. **a** Mitochondrial oxygen consumption rate (OCR) measurement by Seahorse bioenergetics revealed expression of WT or mitochondrial aSYN mitigated mitochondrial respiration function of LUHMES cells, but not in the EGFP controls. **b** LUHMES cell total ATP levels were measured following 72 h of AAV2 viral infection. Cells expressing WT and mitochondrial aSYN showed significant reduction in ATP levels compared to control (data are given as mean ± SEM; *n* = 3; ***p < 0.001 to control, ^#^*p* < 0.05 to aSYN ANOVA, Scheffe’s test. **c**, **e** Representative epifluorescence microscopic images and quantification of LUHMES cells stained with red fluorescent MitoSOX dye. Quantification of MitoSOX-positive cells detected a significant increase in mitochondrial ROS in cells expressing either WT-aSYN or mitochondria-targeted aSYN compared to EGFP controls (data are given as mean ± SEM; *n* = 3; ***p < 0.001 to EGFP control, ANOVA, Scheffe’s test). **d**, **f** Representative epifluorescence microscopic images and quantification of LUHMES cells stained with a red fluorescent TMRE dye. Quantification of TMRE-positive cells identified severe loss of mitochondrial membrane potential in both WT and mitochondrial aSYN-expressing cells compared to EGFP controls (data are given as mean ± SEM; *n* = 3; ***p < 0.001; **p < 0.01 to EGFP control, ANOVA, Scheffe’s test)

Considering the effect of aSYN on the structural integrity of mitochondria, we explored the functional consequences of WT and mito-aSYN overexpression in the dopaminergic neurons. We conducted real-time analysis of mitochondrial respiration function using the Seahorse XFe96 analyser to measure oxygen consumption rate (OCR). Neurons expressing either WT-aSYN or mito-aSYN showed a severe decrease of the mitochondrial basal respiration compared to EGFP controls (Fig. 2a). We also observed a moderate decrease of the maximum respiration in the controls expressing mito-EGFP (Fig. 2a). However, effects of EGFP overexpression on mitochondrial parameters were always moderate and not as pronounced as in cells overexpressing aSYN variants. Further, aSYN variants expressing neurons showed a significant decline of ATP levels compared to naive or EGFP controls (Fig. 2b). Notably, neurons expressing mito-aSYN showed enhanced ATP level loss in comparison to WT-aSYN-expressing neurons (Fig. 2b) implicating stronger toxicity of this mitochondria-targeted variant as observed in the ultrastructural analysis. To show that the differences detected in OCR (Fig. 2a) and cellular ATP status (Fig. 2b) were specific consequences of aSYN toxicity and not linked to increased cell mortality, we determined the cell viability at different time intervals and counted pyknotic nuclei in all the groups. We observed significant LDH release in the aSYN-expressing cells only after 72 h and the remaining conditions at different time points did not altered, indicating that the cells were in the process of cell death (Supplementary Fig. 3a). Since pyknotic nuclei are a hallmark of apoptosis, we observed significant increase in the number of pyknotic nuclei only in the aSYN- and mito-aSYN-expressing neurons after 72 h of viral infection (Supplementary Fig. 3b). Therefore, all the experiments performed in this study were after 72 h of viral infection.

It is established that ROS significantly contribute to mitochondrial damage and death signalling in neurons^29,33^. This applies for aSYN toxicity, where an accumulation of aSYN was linked to ROS formation mediating the neurotoxic effects in model systems of PD^34^. Hence, we examined the influence of aSYN on the generation of mitochondrial ROS in the LUHMES neurons by staining with the red fluorescent MitoSOX dye. Neurons expressing either WT or mito-aSYN showed a massive increase in mitochondrial ROS compared to the EGFP controls (Fig. 2c, e). These results indicated that aSYN overexpression induced mitochondrial ROS formation in the LUHMES neurons, and the mitochondrial localisation of aSYN was sufficient to induce such ROS formation in the organelles. Next, we examined the mitochondrial membrane potential by staining the LUHMES neurons with the red fluorescent TMRE dye. LUHMES neurons overexpressing either form of aSYN displayed diminished TMRE fluorescence intensity compared to the EGFP controls, indicating that aSYN induced a loss of the mitochondrial membrane potential in the dopaminergic neurons (Fig. 2d, f).

### Enhanced cell death in LUHMES and primary cortical neurons expressing mitochondria-targeted aSYN

**Fig. 3:**
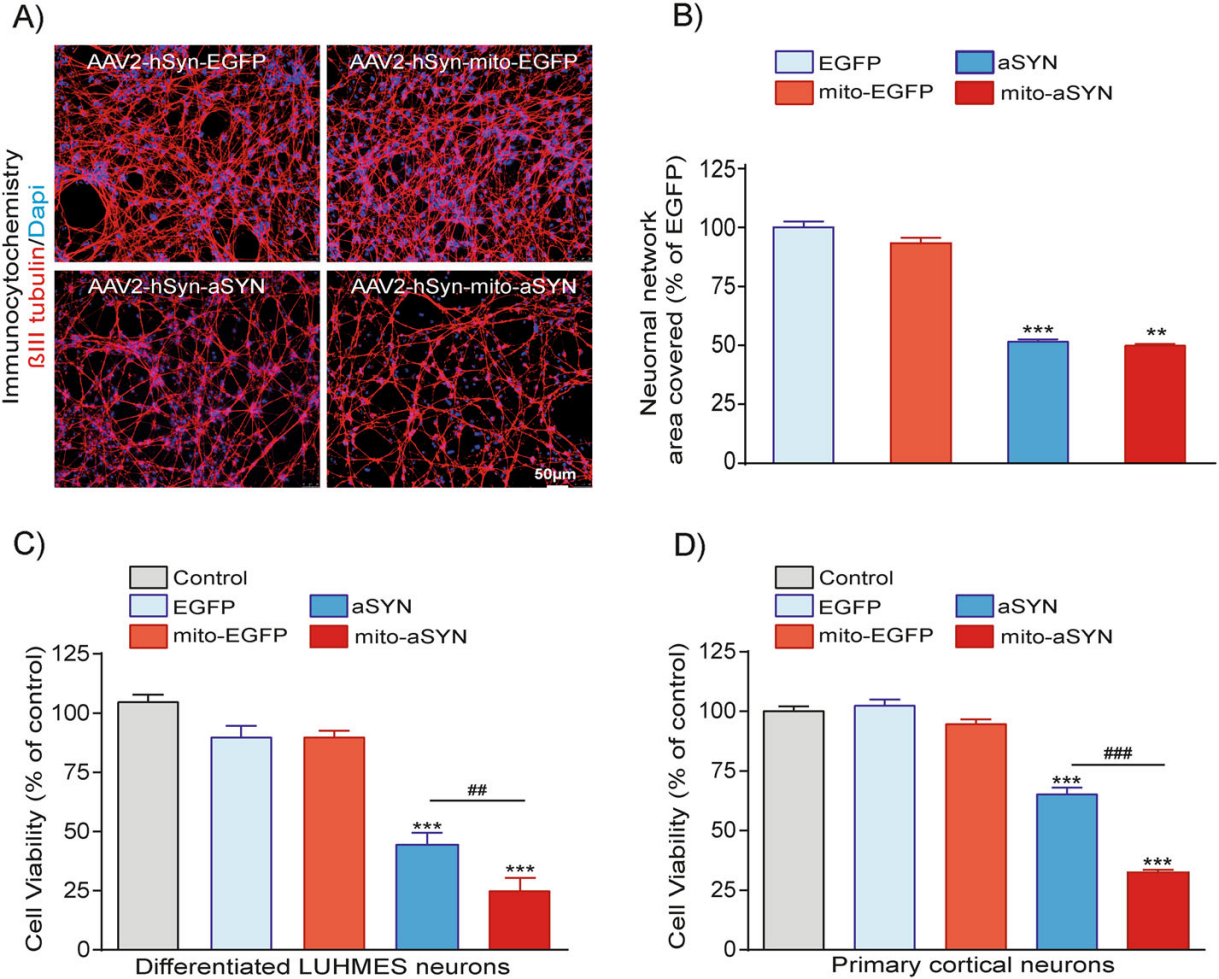
Enhanced neurotoxicity of mitochondria-targeted aSYN in LUHMES and primary rat cortical neurons. **a**, **b** Visualisation of neuronal network degeneration by DAPI (blue) and β3-tubulin (red) immunostaining of differentiated human dopaminergic neurons expressing either WT or mitochondria-targeted aSYN and EGFP control vectors. The degree of neuronal network degeneration is evident in aSYN variants expressing neurons compared to EGFP controls. The degree of neuronal network disintegration was quantified using the Image J software and the intensity levels were plotted (scale bar, 50 µm). (Data are given as mean ± SEM; *n* = 4; ***p < 0.001; **p < 0.01 to EGFP ANOVA–Scheffé’s test.) **c**, **d** MTT cell viability assays confirming both WT-aSYN and mitochondria-targeted aSYN induced significant cell death in LUHMES and primary cortical neurons compared to EGFP controls. Neurons expressing mitochondria-targeted aSYN depicted enhanced cell death compared to WT-aSYN. (Data are given as mean ± SEM; *n* = 8; ***p < 0.001 to controls; ^###^*p* < 0.001; ^##^*p* < 0.01 to aSYN ANOVA–Scheffé’s test)

We investigated the toxicity of the aSYN variants at the level of neuronal network degeneration using β3-tubulin immunostaining and MTT cell viability assay. LUHMES neurons expressing EGFP variants displayed a dense neuronal network (Fig. 3a). In contrast, neurons expressing mito-aSYN or WT-aSYN showed a severely degenerated neuronal network compared to the EGFP controls (Fig. 3a). Quantification of neuronal network confirmed significant network degeneration in neurons expressing WT-aSYN or mito-aSYN (Fig. 3b). Similarly, we observed a significant loss of cytoskeletal tubulin and actin protein levels after transduction of either form of the aSYN as verified by Western blotting (Fig. 1c). Cell viability assays confirmed that LUHMES or primary cortical neurons expressing mitochondria-targeted or WT-aSYN underwent significant cell death compared to the EGFP controls (Fig. 3c, d). These results confirmed that the mito-aSYN variant was more toxic to the neurons than the WT-aSYN.

These findings demonstrate detrimental effects of aSYN on the structural integrity and functional homeostasis of mitochondria with according effects on bioenergetic parameters in human dopaminergic neurons. Mitochondrial localisation of aSYN and according mitochondrial disruption are sufficient to mediate neurotoxicity. Thus, identifying regulators connecting aSYN accumulation and mitochondrial disintegration may provide effective strategies against aSYN neurotoxicity and PD pathology.

### aSYN-induced cell death is mediated via caspase activation in LUHMES neurons

**Fig. 4:**
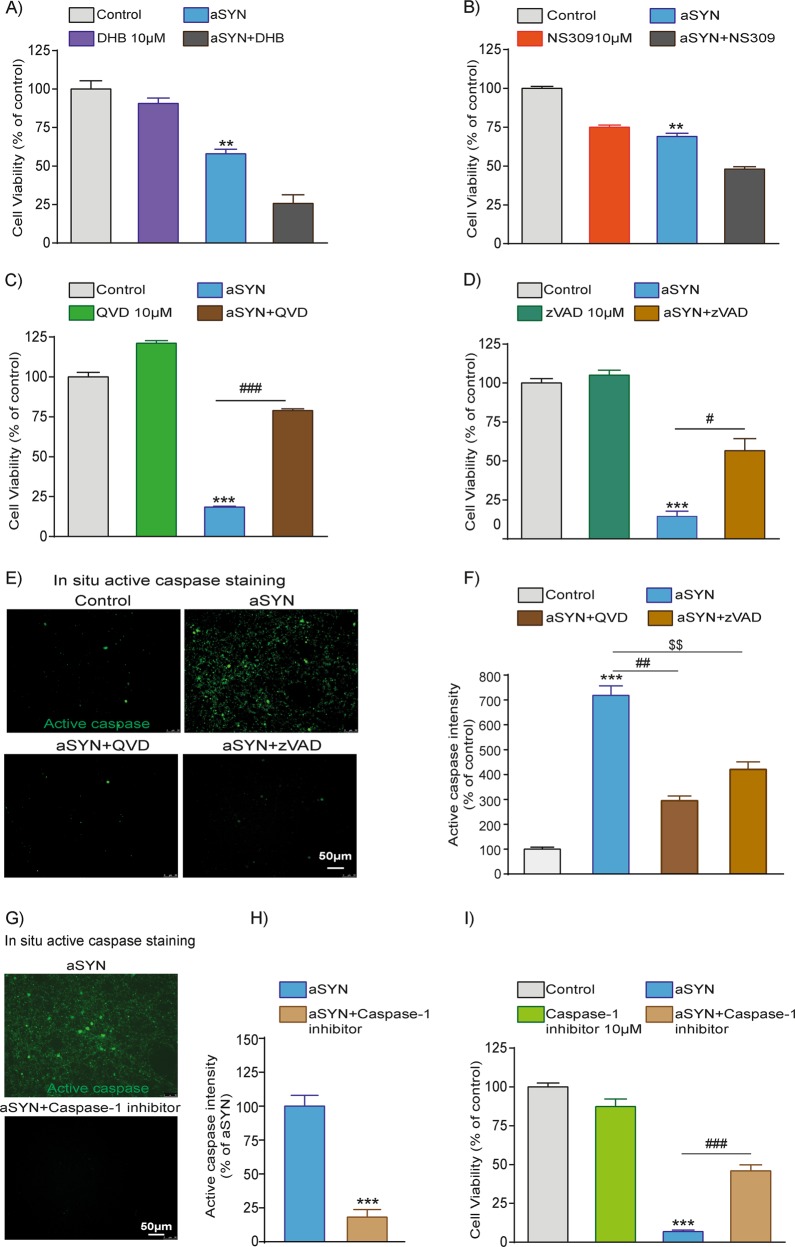
Inhibition of caspases prevented aSYN-induced LUHMES neuronal death, but not the iron chelator DHB or SK2 channel activator NS309. **a**, **b** Inhibition of prolyl hydroxylases (PHD’s) by DHB 10 µM (ethyl-3,4-dihydroxybenzoate) or SK2 channel activator NS309 10 µM failed to inhibit the LUHMES cell death-induced by WT-aSYN as analysed by MTT cell viability assays. (Data are given as mean ± SEM; *n* = 3; **p < 0.01 to control, ANOVA, Scheffe’s test.). **c**, **d** MTT cell viability assays show inhibition of caspases with 10 µM of broad-spectrum caspase inhibitors QVD or zVAD-FMK significantly abolished WT-aSYN-induced LUHMES neuronal death. (Data are given as mean ± SEM; *n* = 4; ***p < 0.001 to control; ^###^*p* < 0.001, ^#^*p* < 0.05 to aSYN, ANOVA, Scheffe’s test.) **e**, **f** Representative images and quantification of green fluorescent active caspases in aSYN-expressing cells and treatment of 10 µM of QVD or zVAD-FMK prevented WT-aSYN-induced caspase activation. (Data are given as mean ± SEM; *n* = 3; ***p < 0.001 control, ^##^*p* < 0.01; ^$$^*p* < 0.01 to aSYN, ANOVA, Scheffe’s test.) **g**, **h** Representative images and quantification of green fluorescent active caspases in aSYN-expressing cells and treatment of 10 µM of caspase-1 inhibitor prevented WT-aSYN-induced caspase activation. (Data are given as mean ± SEM; *n* = 3; ***p < 0.001 aSYN, ANOVA, Scheffe’s test.) **i** Cell viability assays showed inhibition of caspase-1 prevented WT-aSYN-induced LUHMES cell death (Data are given as mean ± SEM; *n* = 3; ***p < 0.001 to control; ^###^*p* < 0.001 to aSYN, ANOVA, Scheffe’s test.)

To prevent the WT-aSYN-induced neurodegeneration, we tested potential drug candidates based on our previous observations (Supplement Table 1). We previously demonstrated the neuroprotective role of HIF-PHD inhibitor, DHB, in models of glutamate-induced oxidative cell death^35,36^. The small conductance Ca^2+^-activated K^+^ channels (SK channel) activator NS309 was previously shown to prevent rotenone-induced oxidative cell death in LUHMES neurons^28^. We tested the neuroprotective ability of DHB and NS309 in LUHMES model system of aSYN neurotoxicity. Neither of the compounds was able to show significant protective effect on aSYN-mediated neurodegeneration in the LUHMES neurons (Fig. 4a, b).

Several observations suggest an involvement of caspases in aSYN-mediated cell death^37–39^. We first tested whether activation of caspases occurs in WT-aSYN-expressing LUHMES neurons using FITC-labelled zVAD-FMK fluorescence analysis. We observed a profound activation of caspases upon aSYN expression as shown by FITC staining’s and quantification of fluorescent intensities (Fig. 4e, f). Then, we tested the neuroprotective role of a broad caspase inhibitor QVD. LUHMES neurons expressing WT-aSYN treated with QVD (10 µM) provided significant protection against aSYN toxicity shown by MTT assays (Fig. 4c). To confirm the protective role of caspase inhibition in this model, we applied another pan-caspase inhibitor, zVAD-FMK (Fig. 4d). Again, the cytotoxicity of WT-aSYN in LUHMES neurons was abolished by zVAD-FMK supporting the role of caspases in aSYN-mediated cytotoxicity in LUHMES neurons.

Using broad range caspase inhibitors, we unveiled caspases are involved in aSYN toxicity. However, which specific caspase was involved in aSYN toxicity remained to be resolved. We found that the caspase-1 inhibitor (YVAD-CMK) prevented WT-aSYN-induced caspase activation as shown by in situ staining and fluorescent intensity quantifications of active caspases (Fig. 4g, h). Similarly, the caspase-1 inhibitor also enhanced the viability of WT-aSYN-overexpressing dopaminergic neurons as shown by MTT assay (Fig. 4i).

### Pan-caspase inhibitor QVD rescues mitochondrial function and electrical activity of LUHMES neurons overexpressing aSYN

**Fig. 5:**
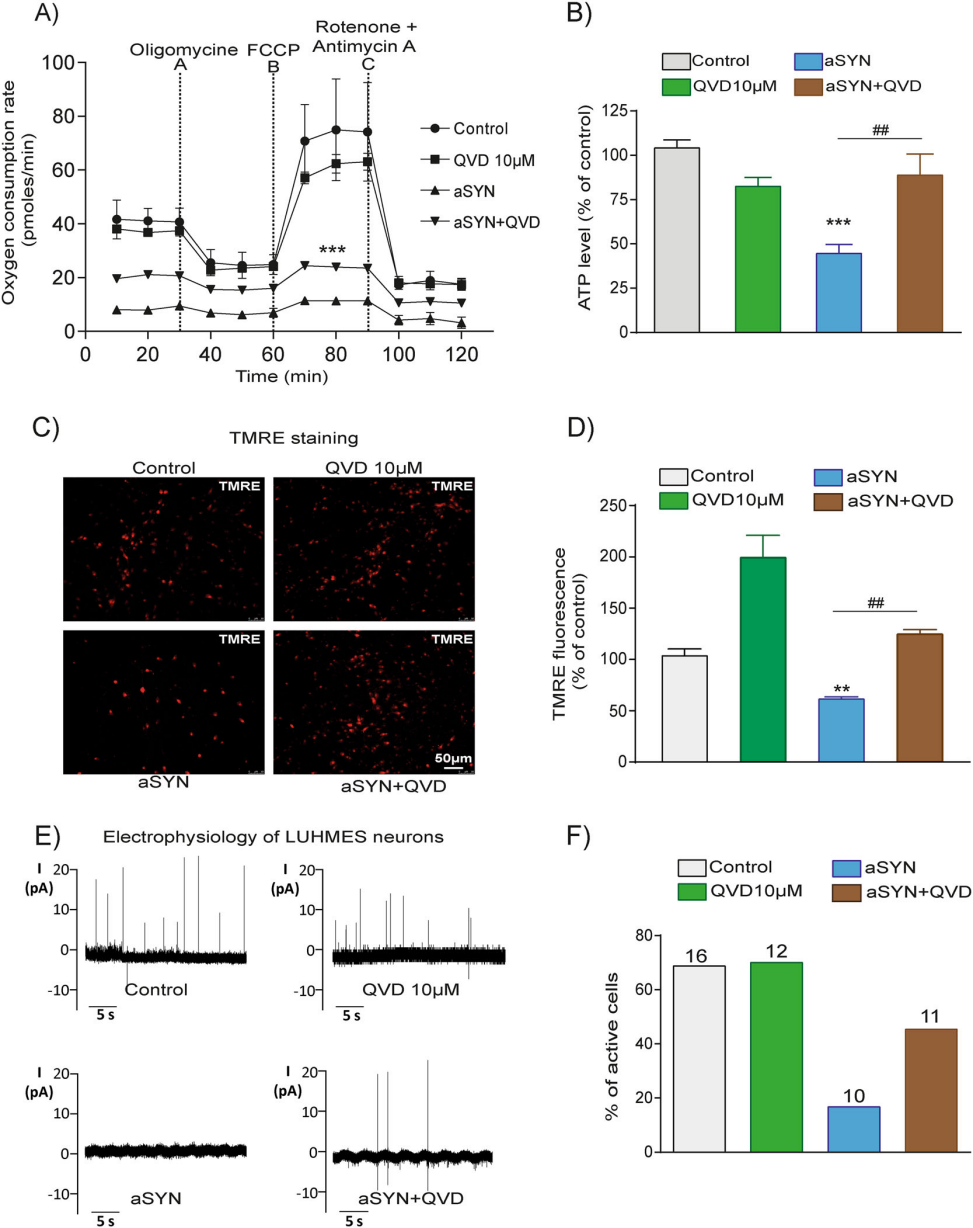
Broad-spectrum caspase inhibitor QVD abolished aSYN-induced mitochondrial dysfunction and preserved electrophysiological properties of LUHMES neurons. **a** Inhibition of caspases by 10 µM QVD protected mitochondrial oxygen consumption rate (OCR) in aSYN-expressing LUHMES neurons (Data are given as mean ± SEM; *n* = 3; ***p < 0.001 to aSYN, ANOVA, Scheffé’s test). QVD alone did not show any impact on mitochondrial respiration. **b** ATP analysis showed WT-aSYN-induced loss of ATP was preserved by 10 µM QVD treatment (Data are given as mean ± SEM; *n* = 3; ***p < 0.001 to control; ^##^*p* < 0.01 to aSYN, ANOVA, Scheffe’s test.) **c**, **d** Representative epifluorescence microscopic images and quantification of fluorescent intensities of TMRE in LUHMES cells stained with a red fluorescent TMRE dye. QVD treatments show more number of TMRE-positive cells compared to WT-aSYN. (Data are given as mean ± SEM; *n* = 3; **p < 0.01 to control; ^##^*p* < 0.01 to aSYN, ANOVA, Scheffe’s test.) **e** Recordings of action potentials obtained in the on**-**cell configuration and **f** the number of active cells per condition are illustrated. WT-aSYN-induced loss of spontaneous membrane potentials was prevented by QVD treatment. The numbers on top of each bar denote the number of cells used for measurement

To further understand the role of caspase inhibition on aSYN-induced LUHMES neuronal cell death, we determined the efficacy of the pan-caspase inhibitor QVD on mitochondrial respiration and network activity. Therefore, we assessed the mitochondrial respiration by measuring the OCR. Treatment with QVD alone did not alter the basal mitochondrial respiration or maximal respiration in comparison to the controls. Neurons expressing WT-aSYN displayed a complete failure of mitochondrial respiration function, and QVD significantly ameliorated the aSYN-induced bioenergetic failure (Fig. 5a). Further, QVD rescued the ATP content in WT-aSYN-overexpressing cells (Fig. 5b). TMRE staining and fluorescence intensity quantifications revealed that QVD also rescued the mitochondrial membrane potential despite WT-aSYN overexpression (Fig. 5c, d).

Differentiated LUHMES dopaminergic neurons form an active neuronal network and generate spontaneous action potentials^40^. To determine the neuroprotective role of QVD against aSYN toxicity at the functional level, we analysed the electrical activity of LUHMES neurons using patch-clamp technology. Voltage clamp recordings in the on-cell configuration revealed spontaneous action potentials or synaptic inputs, with 69% of active cells in controls, and 70% of active cells in QVD-treated controls (Fig. 5e, f). Expression of aSYN drastically reduced the neuronal activity and the number of active neurons to 25%. Treatment of aSYN-expressing neurons with QVD significantly doubled the number of electrically active cells to 50% (Fig. 5e, f). These results suggest that inhibition of caspase activation in aSYN neurotoxicity rescued not only mitochondrial function but also restored electric activity of the neuronal network.

## Discussion

Our study demonstrates a particular role for mitochondrial damage in a model system of aSYN toxicity in human dopaminergic LUHMES neurons. Using a mitochondria-targeted aSYN construct, this study unveils that mitochondrial localisation of aSYN is sufficient to induce severe ultrastructural deformation of the inner mitochondrial cristae membranes, massive swelling and complete loss of respiratory function. Both WT and mito-aSYN overexpression induced a loss of mitochondrial membrane potential and accumulation of mitochondrial ROS. We demonstrate the involvement of caspases with a particular contribution of caspase-1 in aSYN-mediated neurodegeneration.

Several observations in brain tissues of PD patients and transgenic animals expressing aSYN identified the presence of aSYN in the mitochondria^4,17,41–43^. The investigation on differential co-expression identified a PD-specific aSYN transcript with an extended 3′-UTR (aSYN-L). Even though it is believed that aSYN is a synaptic protein involved in neurotransmitter vesical formation, mitochondrial localisation of aSYN-L is attributed to a non-canonical mitochondrial localisation signal^19^. However, direct pathological consequences of mitochondrial aSYN have not been reported so far. Here, we identified exacerbated neuronal toxicity by mitochondria-targeted aSYN in human dopaminergic cells and rodent cortical neurons, indicating the perturbation of mitochondria by aSYN alone is critical for neuronal viability. Concomitantly, both variants of aSYN induced severe loss of the neuronal network, depletion of cytoskeletal actin and tubulin levels and failure LUHMES neuronal firing. These observations are in line with recent reports showing an interaction of aSYN with actin resulted in impaired cytoskeletal dynamics and neuronal network degeneration^44^.

Oxidative stress is a significant contributor of neurodegeneration in models relevant to synucleinopathies and PD. Increased ROS environment promotes cell organelle dysfunction and post-translational modifications of aSYN, such as phosphorylation of serine 129, nitration and ubiquitination, thereby facilitating the formation of toxic oligomers^45–48^. Accordingly, LUHMES neurons expressing WT-aSYN generated high molecular weight aggregates ranging from 50 to 120 kDa of aSYN resistant to denaturing conditions. Interestingly, neurons expressing mito-aSYN did not show prominent high molecular weight aggregates even though mitochondria are one major source of ROS, and this is often further increased in damaged mitochondria. These findings imply that the molecular events contributing to aSYN aggregation mainly occur outside the mitochondria.

Mitochondria are highly dynamic organelles undergoing dynamic fission–fusion processes in a regulated manner. In conditions of oxidative stress, increased mitochondrial fission contributes to mitochondrial damage and failure of cellular energy metabolism^49^. A role for mitochondrial dysfunction in the pathogenesis of PD has long been appreciated. Studies demonstrating mitochondrial accumulation of aSYN in PD brain tissue proposed that aSYN may regulate mitochondrial homeostasis and morphology^4,18,50,51^. Therefore, the role of aSYN on mitochondrial ultrastructural and functional dynamics was further elucidated in the present model of aSYN neurotoxicity in LUHEMS neurons. TEM analysis of dopaminergic neurons overexpressing WT-aSYN revealed many fragmented mitochondria displaying less and malformed cristae in the mitochondria.

Even though aSYN constitutes 1% of the total protein present in neurons and lacks a canonical mitochondrial localisation signal, previous studies proposed essential mechanisms contributing to mitochondrial translocation of aSYN^41–43,52,53^. Electron paramagnetic resonance spectroscopy studies revealed that aSYN interacts explicitly with cardiolipin containing inner mitochondrial membranes^54^. However, the impact of mitochondrial aSYN translocation remained unclear. Using a mito-aSYN construct, we exposed complete distortion of inner cristae structure and swelling of the mitochondria. Intriguingly, the mitochondrial inner cristae membranes were reorganised into small circular structures and were padded at the perimeter of the enlarged mitochondria of the dopaminergic neurons. These circular structures may resemble synaptic vesicles present at the nerve terminals. Due to the amphipathic nature of the N-terminal of the protein, aSYN adopts an extended amphipathic alpha-helix conformation upon binding with lipid bilayers. Thereby, aSYN can remodel the lipid bilayer to a rounded membrane resulting in highly curved, vesicle-like structures^55,56^. In this context, our TEM observations in mitochondria-targeted aSYN-expressing neurons provide direct evidence of membrane remodelling by aSYN in the targeted mitochondria.

The human brain is a highly energy-consuming organ, and, therefore, mitochondrial dysfunction and impaired energy metabolism are major hallmarks of neurodegenerative diseases. Studies in PD patient’s brains suggested that accumulation of aSYN in the mitochondria impaired the function of the mitochondrial complex I^4^. We found a failure of mitochondrial bioenergetics in LUHMES neurons overexpressing mito-aSYN or WT-aSYN. Consequently, aSYN expression significantly decreased the cellular ATP levels indicating a direct impact of aSYN modulation on the cellular energy status. The data presented for mitochondrial respiration (OCR) and ATP measurements represent endpoint data, reflecting the remaining mitochondrial function and overall ATP production in the surviving cells. The effects of aSYN may thus be over-estimated, because fewer cells contribute to these endpoints (Supplementary Fig 3a). However, the bioenergetic indices, like ATP production calculated from basal respiration levels, the different respiratory states and maximal respiration over basal respiration are relative values, which also confirm reduced mitochondrial function in the surviving cells exposed to aSYN. Notably, in cells treated with caspase inhibitors, ATP levels were rescued (Fig. 5b), whereas mitochondrial dysfunction was only partly attenuated (Fig. 5a), suggesting that mitochondrial respiration partly contributed to the energy supply in the protected cells. In models of oxidative stress-induced cell death, we previously documented that mitochondrial dysfunction was associated with increased mitochondrial ROS formation and loss of mitochondrial membrane potential^49^. Similarly, aSYN-induced mitochondrial dysfunction enhanced mitochondrial ROS levels and disruption of the mitochondrial membrane potential leading to dopaminergic neurodegeneration.

In neurodegenerative diseases, the underlying progressive neuronal cell death is considered as a highly regulated process. Reduced glutathione levels, enhanced lipid peroxidation and mitochondrial demise promote an increased pool of labile iron in the cytosol contributing in iron-dependent non-apoptotic PCD coined as ferroptosis. Our observations showed that removal of intracellular labile iron with iron-chelators like DHB ameliorated errastin-induced ferroptotic cell death^49^. In light of these and other observations^10^, we tested the neuroprotective efficacy of the iron chelator DHB in our WT-aSYN model of PD. In contrast to earlier observations in ferroptosis, DHB failed to prevent aSYN toxicity in LUHMES neurons. Similarly, in rotenone-induced oxidative stress model, SK channel activation by NS309 provided neuroprotection in LUHMES cells^28^. However, NS309 did not prevent the WT-aSYN-induced LUHMES neurodegeneration, suggesting that the pathways involved in complex I targeting toxin-mediated cell death may differ in dopaminergic neurons.

Loss of mitochondrial integrity is considered as a point of no return in paradigms of cell death. Compromised mitochondria release cytochrome *c* into the cytoplasm, thereby inducing the activation of caspases. In mammals, ten caspases are classified based on their primary role, such as initiator caspase-2, -8, -9, and -10, or executioner caspase-3, -6 and -7, and caspase-1, -4 (in humans) and -5 are associated with inflammatory responses^57^. Caspases are also involved in cell proliferation, cellular remodelling cell fate determination and immune responses^58^. Studies in PD patient brains and animal models of PD presented a positive correlation of activated caspase-1, -3, -8 and -9 in dying dopaminergic neurons^59–61^. Moreover, studies in hippocampal neuronal cells (HT22) demonstrated that extracellular aSYN induces caspase-3 activation and apoptosis^13^. Here, we found significant caspase activation in WT-aSYN-expressing dopaminergic LUHMES neurons. Inhibition of caspases with the pan-caspase inhibitors QVD or zVAD-FMK significantly ameliorated aSYN toxicity. Notably, caspase inhibition improved not only mitochondrial respiratory function and rescued cellular ATP levels but also enhanced electrophysiological properties of aSYN-expressing neurons. Similarly, Vekrellis et al.^38^ reported that inhibition of caspases abrogated aSYN-induced mitochondrial cytochrome *c* release and caspase-mediated SHSY-5Y neuroblastoma cells death.

Collectively, these results implicate the possible involvement of caspases in aSYN toxicity upstream of mitochondrial damage such as caspase-1, -2 or -8^62^. In fact, we found that inhibition of caspase-1 ameliorated aSYN-induced neurodegeneration. Caspase-1 is known as NLRP3 inflammasome-activating or interleukin-converting enzyme and is regarded as a key mediator of inflammatory process. In addition, caspase-1-mediated cell death is known as pyroptosis in macrophages following infection. Furthermore, caspase-1 was shown to induce apoptosis involving Bid (BH3-interacting domain) and mitochondrial-dependent activation of caspase-3 in stroke models^63^. Recent findings reveal that involvement of caspase-1 activated NLRP3 inflammasome via mitochondrial ROS production induced profound neurodegeneration in MPTP-treated microglia-neuron co-culture model^64^. Concomitantly, inhibition of caspases was effective in preventing the aSYN toxicity despite the marked impairment of mitochondrial structure, implying unknown caspase-dependent mechanisms upstream of mitochondrial pathways of aSYN toxicity. Even though the mode of cell death observed is caspase-dependent, we did not detect typical apoptotic nuclear morphology in TEM suggesting an involvement of a tapestry of mechanisms in aSYN-induced dopaminergic neurodegeneration.

Altogether, we unveiled the fatal remodelling of mitochondrial cristae membranes by aSYN leading to an extensive swelling and bioenergetic failure of the mitochondria in human dopaminergic neurons. We demonstrated that caspase inhibition reversed aSYN-induced mitochondrial dysfunction and neuronal death, suggesting that caspase inhibition and specifically inhibition of caspase-1 might be an effective therapeutic strategy in synucleinopathies including PD.

## Supplementary information

Supplementary Table 1

Supplementary figure 1

Supplementary figure 2

Supplementary figure 3
