# Supplementary material for: Mitochondrial damage by α-synuclein causes cell death in human dopaminergic neurons
Source: Cell Death Dis. 2019 Nov 14;10(11):865. doi: 10.1038/s41419-019-2091-2 (PMC6856124; doi:10.1038/s41419-019-2091-2)
Supplement: Supplementary file 1 — Supplementary Table 1 [file 41419_2019_2091_MOESM1_ESM.docx]

| **Drugs** | **Applied concentration** | **% change in cell viability**  **(WT aSYN *vs* WTaSYN+drug)** |
| --- | --- | --- |
| DHB (Iron chelator) | 10µM | -32.22 % |
| NS309 (SK channel activator) | 10µM | -28.09 % |
| ICE inhibitor II (Caspase 1 inhibitor) | 10µM | +39.01 % |
| zVAD (Broad range caspase inhibitor) | 10µM | +42.08 % |
| QVD (Broad range caspase inhibitor) | 10µM | +60.39 % |

Supplement Table 1. Potential drug candidates of cell death inhibitors tested against WT aSYN toxicity in LUHMES neurons
