# Supplementary figures and images for: Mitochondrial damage by α-synuclein causes cell death in human dopaminergic neurons
Source: Cell Death Dis. 2019 Nov 14;10(11):865. doi: 10.1038/s41419-019-2091-2 (PMC6856124; doi:10.1038/s41419-019-2091-2)

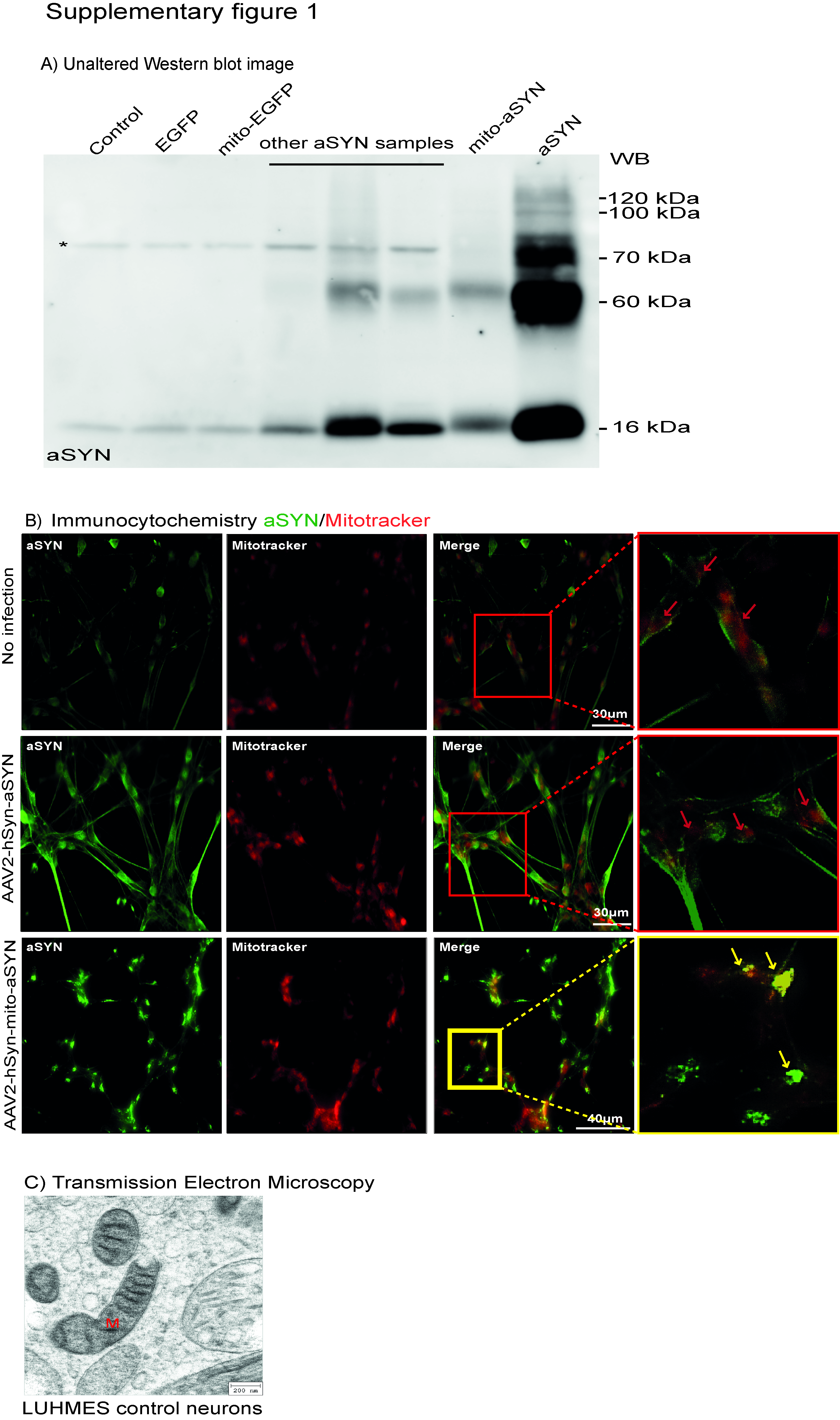

Supplement: Supplementary file 2 — Supplementary figure 1 [file 41419_2019_2091_MOESM2_ESM.tif]

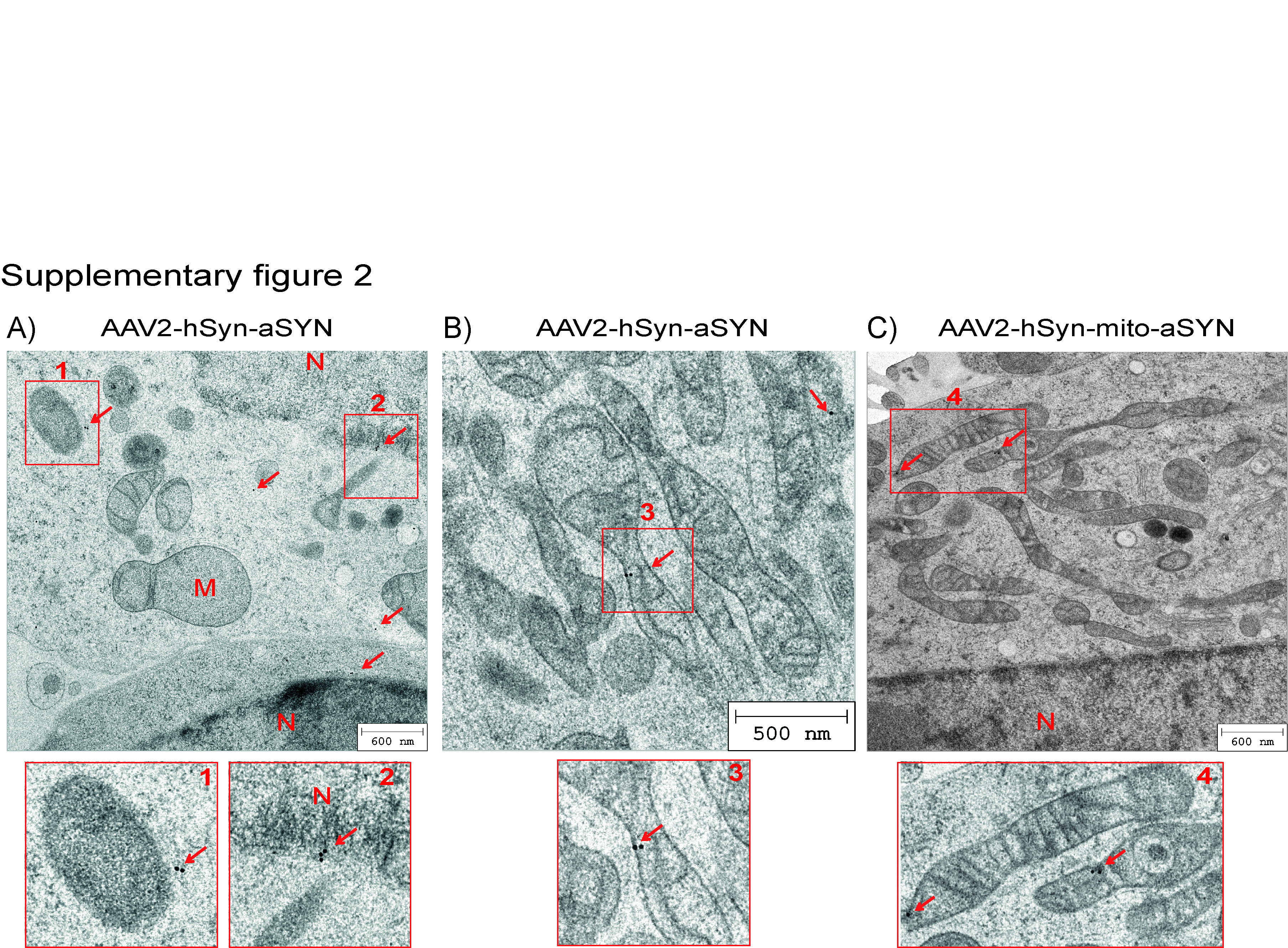

Supplement: Supplementary file 3 — Supplementary figure 2 [file 41419_2019_2091_MOESM3_ESM.tif]

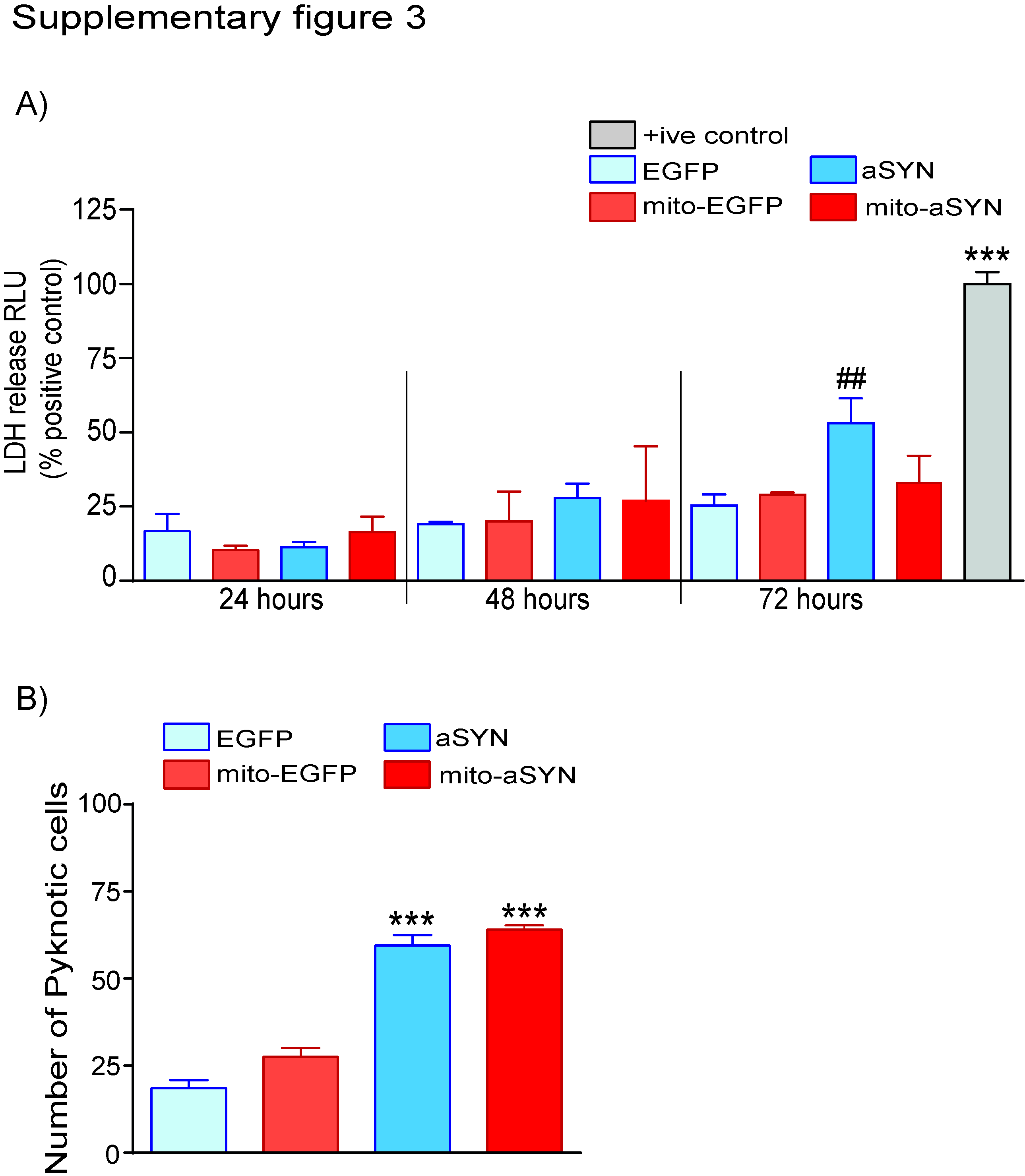

Supplement: Supplementary file 4 — Supplementary figure 3 [file 41419_2019_2091_MOESM4_ESM.tif]
